# Four-Month-Old Infants’ Long-Term Memory for a Stressful Social Event

**DOI:** 10.1371/journal.pone.0082277

**Published:** 2013-12-12

**Authors:** Rosario Montirosso, Ed Tronick, Francesco Morandi, Francesca Ciceri, Renato Borgatti

**Affiliations:** 1 Centre 0–3 for the Study of Social Emotional Development of the at Risk Infant, Scientific Institute, IRCCS Eugenio Medea, Bosisio Parini (Lecco), Italy; 2 Department of Psychology, University of Massachusetts, Boston, Massachusetts, United States of America; 3 Pediatric Unit, Sacra Famiglia Hospital, Erba (Como) Italy; 4 Biology Laboratory, Scientific Institute, IRCCS Eugenio Medea, Bosisio Parini (Lecco), Italy; 5 Neuropsychiatry and Neurorehabilitation Unit, Scientific Institute, IRCCS Eugenio Medea, Bosisio Parini (Lecco), Italy; Università di Parma, Italy

## Abstract

Infants clearly show an early capacity for memory for inanimate emotionally neutral events. However, their memory for social stress events has received far less attention. The aim of the study was to investigate infants’ memory for a stressful social event (i.e., maternal unresponsiveness during the Still-Face paradigm) after a 15-day recall interval using changes in behavioral responses and salivary post-stress cortisol reactivity as measures of memory. Thirty-seven infants were exposed to social stress two times (experimental condition); the first time when they were 4 months of age and second exposure after a 2 week interval. Infants in the control condition (N = 37) were exposed to social stress just one time, at the age corresponding to the second exposure for infants in the experimental condition (4 months plus 2 weeks). Given individual differences in infants’ reactivity to social stress events, we categorized infants as *increasers* or *decreasers* based on their cortisol reactivity after their initial exposure to the stress of the maternal still-face. Infants in the experimental condition, both *increasers* and *decreasers*, showed a significant change in cortisol response after the second exposure to the maternal still-face, though change was different for each reactivity group. In contrast, age-matched infants with no prior exposure to the maternal still-face showed similar post-stress cortisol reactivity to the reactivity of the experimental infants at their first exposure. There were no behavioral differences between *increasers* and *decreasers* during the Still-Face paradigm and exposures to the social stress. Thus differences between the experimental and control groups’ post-stress cortisol reactivity was associated with the experimental group having previous experience with the social stress. These findings indicate long-term memory for social stress in infants as young as 4 months of age.

## Introduction

There is considerable evidence that in human infants memory capacity emerges early in the first year of life [1], [2], [3]. Infants evidence operant conditioning (e.g., using the mobile conjugate reinforcement) as early as 2 months of age with retention interval delays up to one day, which gradually increases to 8 weeks for 12-month-olds [4]. Three- and six-month-old infants recognize static images of faces after retention intervals of 24 hours [5], nine month-old infants evidence retention of the individual actions of multi-step sequences over delays of 1 month [6] and six-month-olds exhibit deferred imitation after a 24-h delay [7]. Moreover, recent evidence suggests that the social context affects long-term memory ability. To investigate the effects of the degree of joint attention (high *vs.* low) at initial encoding of objects on long-term retention processes in 4-month-old infants, event-related potentials in response to memory test procedure were used to assess the immediate recognition and at a 1-week delay. Pb amplitude and latency in delayed recognition phase differed between low and high joint attention condition, suggesting that social cues had an influence on how objects were recognized after a 1-week delay [8]. Thus while there is still much debate as to the relative development of implicit versus explicit memory [9], taken together the data from typically-developing infants converge to suggest that during the first months of life infants’ long-term memory capacity increases and the social context can affect memory.

Nevertheless, it is important to note that most infant memory research has studied emotionally neutral events and non-social cognition (e.g., learning tasks), whereas infant memory for social events has not been extensively studied. Bornstein, Arterberry and Mash [10] found evidence suggestive of memory for a mildly negative emotional event at 20 months of age which was experienced at 5 months of age. This scarcity of research of infants’ memory for social stress is surprising given the view that infants’ early social and stressful experiences, including their early interactions, contribute critically to their subsequent socio-emotional development and memorial capacities [11], [12]. For example, a recent studies have found that six month-old infants showed an anticipatory response associated to moderate social stress 24 hours later [13] and that children experiencing neglect and/or emotional maltreatment and low cortisol had heightened false recognition memory [14]. In the current study, using behavioral responses and salivary post-stress cortisol reactivity, we examined whether four-month old infants’ memory for a stressful social event (i.e., maternal still-face) was preserved after a 15-day recall interval when they were again exposed to same event. To evaluate if there was an memory for the social stress, we compared the infants’ responses in the experimental group to those of age-matched control infants with no prior exposure to the same stressful event.

It is well-established that stress-induced cortisol release affects memory, however the direction of this effect can vary [15]. Some studies have found that stress enhances memory performance, whereas others have found a detrimental effect of stress on memory. On the one hand, in some studies of animals and adults, mild to moderate levels of adrenocortical activation during or shortly after a stressor enhance memory for the stressor [16] and glucocorticoids administered after exposure to emotionally arousing experiences enhance the consolidation of long-term memories of these experiences [17]. On the other hand, high levels of adrenocortical activation are associated with poor memory and attention [18] and de Quervain, Roozendaal and Roozendaal [19] found that cortisol impaired retrieval of neutral words learned 24 h earlier, while having no effect on the initial learning or on the consolidation of the memory for the words.

Previous studies have also documented large individual differences in the hypothalamic-pituitary-adrenal (HPA) axis reactivity in response to social stress suggesting that individuals who react to a social stress with cortisol elevation (i.e., high responders) have significantly impaired social memory performance [20]. In a study using psychosocial stressors, adult subjects showing a pronounced cortisol increase in response to the stressor performed poorer in declarative memory tasks than subjects showing only a mild response [21]. Takahashi and colleagues [22] have confirmed that social stress-induced cortisol elevation acutely impairs social memory and high stress responders also appear to be impaired in working memory functions [23].

Infant studies examining the associations between variation in circulating levels of cortisol and memory have found inconsistent results. Three month-old infants with an increase in cortisol concentration during the learning phase of a task had better memory after 24 hours than infants who had decreased cortisol levels [24]. In contrast, three month-old infants showing a decline in cortisol levels between phases before and after a learning phase exhibited better memory than infants whose cortisol increased [25]. To date, with one exception, which did not examine individual differences in physiological reactivity [13], the research of the effects of stress hormones on infant social memory has been neglected. Thus, the question of how individual differences in cortisol reactivity are associated with infant memory for emotional events remains open.

Infants show a stress response during stressful social interactions, such as a brief relational disruption (i.e., FFSF paradigm, [26]. During the FFSF infants engage in normal face-to-face interactions with their caregiver and also confront the perturbation of the caregiver remaining poker-faced and unresponsive (i.e., maternal still-face episode). Infant responses to the mothers’ lack of responsivity are marked by increases in negative affect, a drop in positive affect and in social monitoring [27], [28]. Furthermore, some infants show clear physiological stress reactions with a post-FFSF elevation of the salivary cortisol concentration [29], [30], [31], [32], whereas other studies find a decrease in post-stress cortisol levels [33], [34]. Although, the source of these differences are not clear [35], these findings reveal early individual differences in HPA axis reactivity [36].

The overall goal of the current study was to examine infants’ memory for a stressful social event using the FFSF paradigm and to take into account individual differences in stress reactivity. Since the HPA axis is only partially linked to behavior [36], [37], we used multiple measures of memory including behavioral responses (i.e., negative emotionality across the FFSF) and salivary post-stress cortisol reactivity. To evaluate memory effects we compared experimental group infants’ memory for the maternal unavailability after a 15-day recall interval to that of age-matched control infants with no prior exposure to the FFSF. We expected that infants in the experimental condition would exhibit evidence for memory for the FFSF both at physiological and behavioral levels compared to age-matched control infants. Importantly, we evaluated how individual differences in infants’ physiologic reactivity (i.e., increased or decreased post-stress cortisol concentrations) at their first exposure to the FFSF were associated with their memory for it at their second exposure. Though tentative because of a lack of previous work, we expected a memory effect which would be affected by individual differences in stress reactivity. That is, if some infants evidenced a pronounced cortisol response to the FFSF, while others respond with a reduction in the post-stress cortisol levels, we expected that their cortisol response to a second exposure of the social stress after a recall interval would also differ. Similarly, we expected that during the second FFSF infants would show a difference in the behavioral engagement (e.g., more or less infant negativity) than at their first exposure to the FFSF and compared to the age-matched control infants.

## Methods

### Participants

Seventy-four 4 month-old infants and their mothers participated in the study. The infants were all healthy full-terms and were recruited from the regular nursery at Pediatric Unit of the Sacra Famiglia Hospital of Erba, Italy. The selection criteria for the infants were: full-term gestation (≥37 weeks), Apgar scores of at least 7 at 1 min and 8 at 5 min, no congenital abnormalities, appropriate weight for gestational age, and uncomplicated prenatal, perinatal and neonatal courses.

The dyads were randomly assigned to the experimental or control condition. For infants in the experimental condition (N = 37) the first exposure to the FFSF took place when the infants were 4 months of age (T1-Exp) and the second exposure was 2 weeks later (T2-Exp). Infants in the control condition (N = 37) were videotaped once in the FFSF, at the age corresponding to the second exposure for infants in the experimental conditions (T2-Ctrl). The inclusion of a control group insured that any changes of infants’ stress hormones in experimental group between the first and second exposure, would be a function of the prior emotional stress of the maternal unresponsiveness rather than the effect of the novelty of the laboratory or other confounds at T1 and T2.

### Procedure

All mothers were told that the study was concerned with infant–caregiver interactive behavior. For the mothers of infants in the experimental condition they were also told the study was concerned with memory. Mothers who expressed an interest in participating in the study were scheduled to visit the laboratory of the Scientific Institute IRCCS Eugenio Medea, when they thought their infant would be awake and alert. To accommodate the schedules of mothers and infants, it was necessary for the testing to be done throughout the day (9∶30 a.m. to 17∶00 p.m.). Upon arrival, informed consent was obtained from the mother, and then basal saliva samples were collected from the infant.

To avoid milk contamination and consistent with previous work [38], no infant was fed prior to collecting the samples across the procedure and mothers were told that their infants could not be fed during the 40-min period after the FFSF procedure. Mothers completed a socio-demographic survey (i.e., infant demographic variables, obstetric characteristics and maternal characteristics), and a brief questionnaire about any recent events that could influence salivary cortisol levels (i.e., last nap and last feeding prior to FFSF onset). Following the completion of the questionnaires a video recording of the mother–infant interaction was made using the FFSF procedure.

### Ethics Statement

The study was approved by the Ethics Committee of the Scientific Institute IRCCS Eugenio Medea and written informed consent was obtained from every mother.

### Face-to-Face-Still-Face Paradigm

Dyads in each group at each visit were videotaped in the double-exposure modification of original FFSF paradigm [26]. This modification of the 3 episode FFSF was used because it has been found to more reliably generate a cortisol response in the infants [29]. The double FFSF has 5 successive 2-minute episodes: 1) Play: the mother plays normally with her infant; 2) Still-Face#1: an experimental perturbation in which the mother is instructed to look at the infant but to keep a neutral facial expression and not respond to her infant; 3) Reunion#1: the mother resumes her normal play interaction; 4) Still-Face#2, and 5) Reunion#2. All FFSF were done in the same room of the laboratory, which was equipped with an infant-seat mounted on a table, an adjustable swivel stool for the caregiver approximately 0.4 m from the infant and adjusted so that her eyes were level with her baby’s eyes, two cameras (one focused on the infant, the other on the caregiver), and a microphone. Signals from the two cameras were edited off-line to produce a single image with simultaneous frontal view of the face, hands, and torso of infant and mother. For analysis purposes, a computerized digital format was created for each infant-mother dyad’s video clip. Additionally, mothers were asked to put on a yellow smock at the beginning of the FFSF at each visit to serve as a unique memory cue and to provide a distinctive memory cue for the infant at T2-Exp. The smock was worn at both visits for the experimental mothers and by the control group mothers at their single visit.

### Infant Behavioral Response

The infants’ behavioral engagement was micro-analytically coded by independent coders masked to experimental condition with the Infant and Caregiver Engagement Phases (ICEP) [39]; using 1 sec. sampling. In the present study a Negative engagement index was used, which described when the infant was negative or protesting. That is, the infant displayed negative facial expressions, (e.g., sadness, distress, crying, or grimacing), complaining, being fussy, crying vocalizations. For each of the five FFSF episodes, the proportion of time in which the infant was in Negative engagement, was obtained by dividing the total time of Negative engagement by the total length of the episode. Coders were trained with a gold standard sample of ten videotapes (agreement >75%). They were masked to group membership and unaware of the cortisol data. Twenty percent of FFSF for all three sessions were also randomly selected and evaluated for agreement between two independent coders. Inter-observer reliability was determined through percentage agreement and Cohen’s kappa. The mean percentage of agreement and Cohen’s kappa were 83% and.75 respectively.

### Salivary Cortisol

Two samples values were collected prior to the FFSF. The first saliva sample was collected shortly after arrival in the lab; the second one was taken approximately 10 minutes later, after briefing the mother. During this period the infant was quietly settled on his or her mother’s lap. Three additional measures were taken 10, 20 and 30 minutes after the end of the FFSF procedure. Salivary cortisol was collected from the infant using a dental roll that was swabbed in the infant’s mouth until it became saturated with saliva. Oral stimulants to increase saliva flow were not used to avoid any problem with the cortisol assay associated with their use [40]. All infants tolerated the saliva sampling across the procedure. Saliva samples were stored at –80°C until assayed in the biology laboratory of IRCSS Medea using the Salimetrics High Sensitivity Salivary Cortisol Enzyme Immunoassay Kit for quantitative determination of salivary cortisol (Salimetrics LLC, State College, PA). Average intra- and inter-assay coefficients of variation were both less than 10%. Cortisol data were examined for outliers (i.e., any value higher/lower than 3 SD from the mean) [41]. In the data for the three groups, there were no scores that met this criterion.

### Time of Day

Given the diurnal variation of cortisol, time of day of the exposure was evaluated. There were significant differences between T1-Exp (M = 12.84; SD = 2.50) and T2-Ctrl (M = 11.37; SD = 1.60), *t*(61.39) = 3.01, *p* = .004, and between T1-Exp and T2-Exp (M = 12.29; SD = 2.42), *t*(36) = 2.44, *p* = .020. Consequently, time of day was considered as a covariate in the cortisol data analyses.

### Data Reduction and Statistical Analysis

To evaluate if there were different maternal and infant characteristics between experimental and control group, *χ* ^2^ (for categorical variables) and independent samples *t*-tests (for continuous variables) were carried. A cortisol reactivity index (CRI) was computed as follows: 1) baseline level was calculated as the mean of the two pre-FFSF cortisol samples; 2) since the raw cortisol values (in µg/dl) in baseline and in the post-FFSF peak were not normally distributed, the raw scores were transformed to log10 scores [42]; and 3) to remove the dependence on the baseline initial value the CRI was computed as a division of post-FFSF peak by baseline. To examine individual differences in physiologic reactivity infants were grouped into *increasers* and *decreasers*: infants who had higher or lower cortisol values post-FFSF compared to pre-FFSF values at their first exposure to the FFSF. Thus, negative CRI values represented a decrease in physiologic reactivity and positive CRI values represented an increase in physiologic reactivity. In the experimental condition there were 22 *increasers* and 15 *decreasers*, while in the control condition there were 20 *increasers* and 17 *decreasers*.

To determine whether infant behavioral response and salivary post-stress cortisol reactivity varied in relation to exposure to FFSF across episodes of the FFSF paradigm, three separate ANOVAs were separately performed with Negative engagement and with CRI as dependent variables. For behavior, 1) to compare infants at their first exposure to FFSF, Physiologic reactivity (*increasers vs*. *decreasers*) and Exposure (T1-Exp *vs.* T2-Ctrl) were entered as between-subjects variables and Episode (Play, Still-Face#1, Reunion#1, Still-Face#2, Reunion#2) as within-subjects factor; 2) to compare infants with prior exposure to the FFSF and age-matched infants in the control group, Physiologic reactivity (*increasers vs. decreasers*) and Exposure (T2-Exp *vs*. T2-Ctrl) were entered as between-subjects variables and Episode (Play, Still-Face#1, Reunion#1, Still-Face#2, Reunion#2) as within-subjects factor; and 3) to compare infants with the first and second exposure to FFSF, Physiologic reactivity (*increasers vs. decreasers*) was entered as the between-subjects factor, Exposure (T1-Exp *vs.* T2-Exp) and Episode (Play, Still-Face#1, Reunion#1, Still-Face#2, Reunion#2) as within-subjects factors. For salivary post-stress cortisol reactivity: 1) to compare infants at their first exposure to FFSF, Physiologic reactivity (*increasers vs*. *decreasers*) and Exposure (T1-Exp *vs.* T2-Ctrl) were entered as between-subjects variables; 2) to compare infants with prior exposure to the FFSF and age-matched infants in the control group, Physiologic reactivity (*increasers vs. decreasers*) and Exposure (T2-Exp *vs*. T2-Ctrl) were entered as between-subjects variables; and 3) to compare infants with the first and second exposure to FFSF, Physiologic reactivity (*increasers vs. decreasers*) was entered as the between-subjects factor and Exposure (T1-Exp *vs.* T2-Exp) as within-subjects factor. Time of day was entered as covariate in these ANOVAs. Pairwise comparisons (Bonferroni-corrected) and *t*-tests were conducted to further investigate significant effects. Effect size was evaluated by using the partial *η* squared (*η* ^2^_ p_). Pearson correlations were used to evaluate the relation between Negative engagement in the FFSF episodes, and CRI. The correlations were calculated separately by Exposure and Physiologic reactivity. All analyses were performed at a significance level *p*≤.05, using SPSS for Windows (version 17, Chicago, IL, USA).

## Results

### Infant and Maternal Characteristics

No differences were found between experimental and control group for infant characteristics, such as gender, birth weight and Apgar. Furthermore, no differences emerged for cortisol-related variables (i.e., last nap and last feeding prior to FFSF onset). There were no differences in maternal age, maternal education levels and socio-economic status between experimental and control group (see Table 1).

**Table 1:** Maternal characteristics of infants group for control condition and experimental one.

	Control condition						Experimental condition					
	*Increasers*		*Decreasers*		*All*		*Increasers*		*Decreasers*		*All*	
	(N = 20; F = 10)		(N = 17; F = 9)		(N = 37)		(N = 22; F = 11)		(N = 15; F = 8)		(N = 37)	
	*Mean*	*SD*	*Mean*	*SD*	*Mean*	*SD*	*Mean*	*SD*	*Mean*	*SD*	*Mean*	*SD*
Age (yrs)	34.30	4.31	34.50	5.21	34.39	4.68	33.72	3.44	34.31	3.58	33.95	3.48
Education (yrs)	14.95	4.01	14.59	3.41	14.78	3.70	13.55	3.39	13.33	3.12	13.46	3.27
Socioeconomic status*	69.00	16.51	64.71	17.00	67.03	16.64	53.64	22.32	52.67	20.83	53.24	21.59

=  Female; *Hollingshead’s classification [43]. Note: F

### Infant Behavioral Response

**Figure 1:**
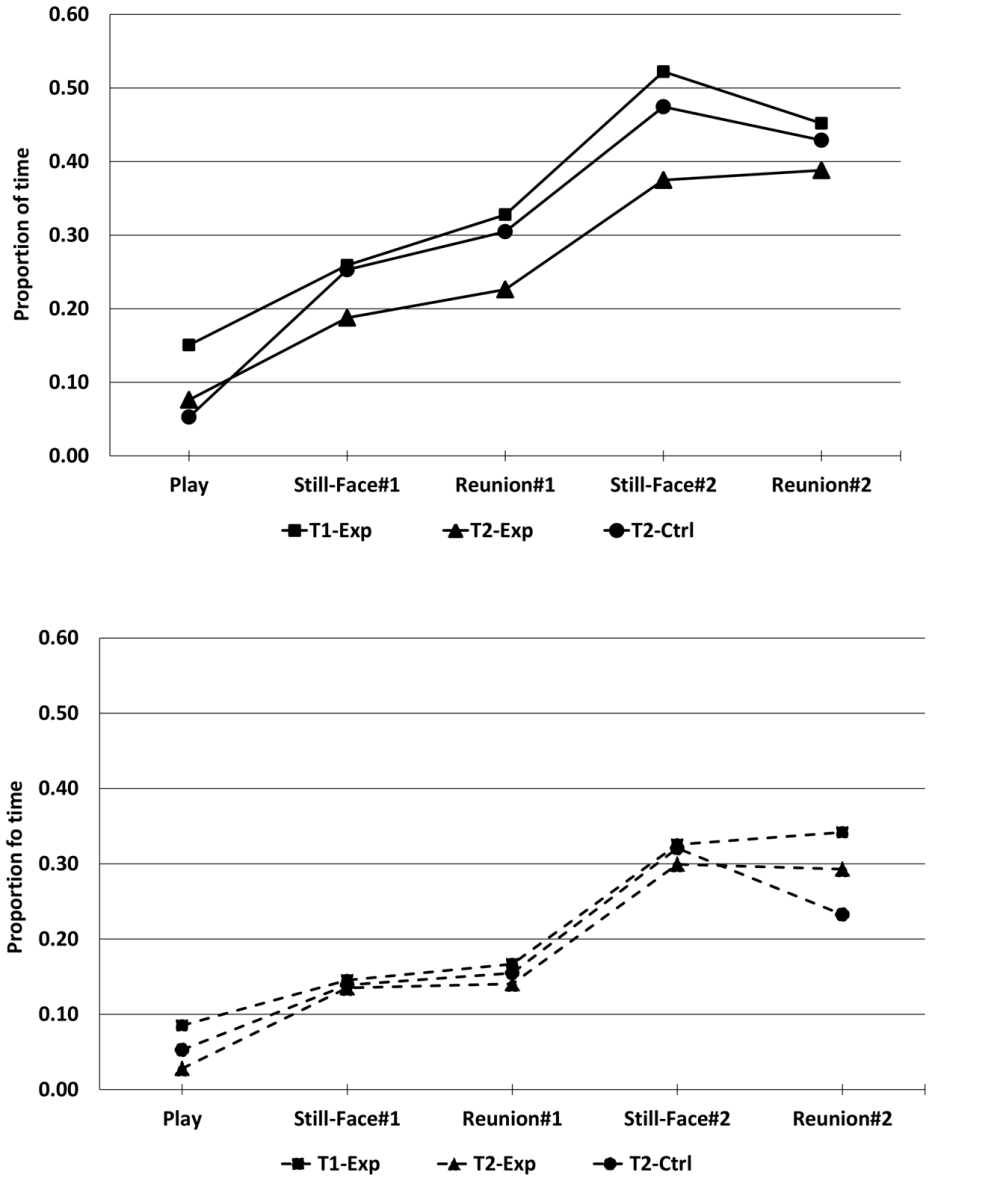
Means of Negative engagement across the FFSF paradigm for *increasers* (black line) and *decreasers* (dotted line) in the first exposure (T1-Exp, 4 months of age) and second exposure (T2-Exp, 4 months +15 days, grey line) of the experimental group and control group (T2-Ctrl, 4 months +15 days).

As shown in Figure 1 Negative engagement gradually increased over the episodes of the FFSF at each exposure (all *F*s ≥18.57, *p*≤.000, η^2^_p_ = .35). However, there were no significant behavioral differences between *increasers* and *decreasers* over episodes or exposures.

### Cortisol Reactivity Index

#### Comparisons relative to the first exposure to FFSF

**Figure 2:**
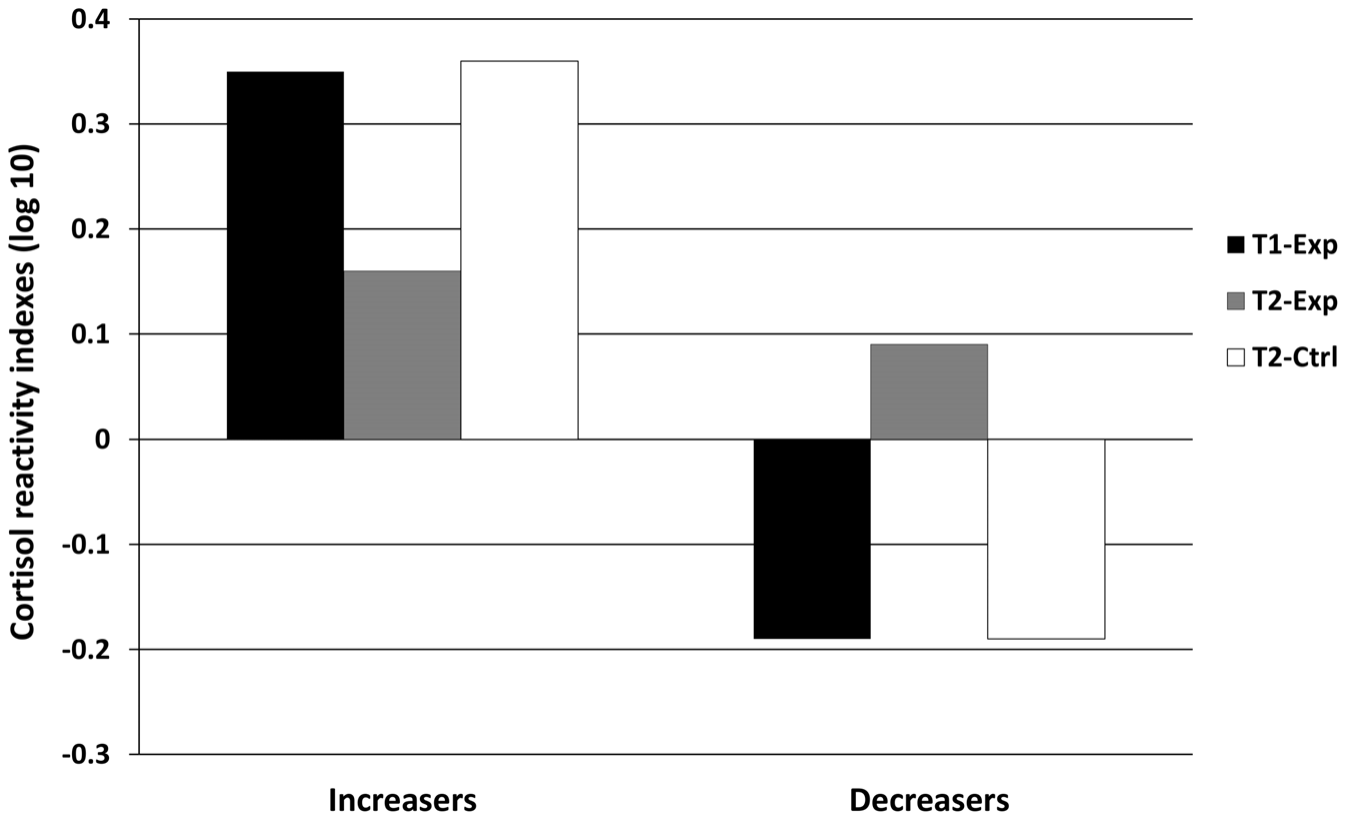
Cortisol reactivity indexes (CRI) and error bars represent standard errors for *increasers* and *decreasers*. Infants in the experimental condition, both *increasers* (N = 22) and *decreasers* (N = 15), showed significant changes in post-stress cortisol concentrations between first (T1-Exp, 4 months of age, black bars) and second exposure (T2-Exp, 4-months +15-days, grey bars). In contrast, age-matched infants with no prior exposure to social stress (T2-Ctrl, 4-months +15-days, white bars), both *increasers* (N = 20) and *decreasers* (N = 17), showed a similar post-stress cortisol response to those exposed the first time to social stress (T1-Exp), but a different pattern when compared with infants with a prior exposure to FFSF (T2-Exp).

Figure 2 shows CRIs for *increasers* and *decreasers* in experimental and control groups. No main effects, interactions or covariate effects were found in CRI between T1-Exp and T2-Ctrl, indicating that both groups had a similar post-stress cortisol response at their first exposure.

#### Comparisons between infants with prior exposure to the FFSF and age-matched infants in the control group

A significant interaction for Exposure x Physiologic reactivity was found, *F*(1,69) = 13.73, *p* = .000, η^2^_p_ = .17, indicating that CRI differed for both *increasers* and *decreasers* between T2-Exp and T2-Ctrl. Follow-up unpaired *t*-tests indicated that *increasers* of T2-Ctrl showed higher CRI than those of the T2-Exp, *t*(40) = 2.34, *p* = .024 and *decreasers* of T2-Ctrl presented lower CRI than those of T2-Exp, *t*(18.6) = −3.01, *p* = .007. Thus the cortisol response was different if infants were previously exposed or not to maternal unresponsiveness, suggesting an effect of the experience. No covariate effects were found.

#### Comparisons between the first and second exposure to FFSF

MANCOVA revealed a significant interaction of Exposure x Physiologic reactivity, *F*(1,33) = 15.83, *p* = .000, η^2^_p_ = .32, indicating that the CRI differed for both *increasers* and *decreasers* between T1-Exp and T2-Exp. Follow-up paired *t*-tests found that from the first to the second exposure, *increasers* showed a post-FFSF cortisol concentration reduction, *t*(21) = 2.56, *p* = .018, and *decreasers* a post-FFSF cortisol concentration elevation, *t*(14) = −2.70, *p* = .017. No covariate effects were found.

### Relation Between Infant Behavioral Response and Cortisol Reactivity Index

There were no significant correlations between Negative engagement and salivary post-stress cortisol reactivity, suggesting a lack of correspondence between behavioral and physiologic reactivity measures during FFSF.

## Discussion

The main aim of the study was to examine whether or not four-month old infants have a memory for a stressful social event (i.e., maternal still-face) after a 15-day recall interval when they were again exposed to same social event. The data support the expectation that infant have long term memory for a social stressor over the recall interval. Infants in the experimental condition showed a change in the cortisol response to social stress after the second exposure. In contrast, age-matched infants with no prior exposure to the maternal still-face showed similar post-stress cortisol reactivity to the response of the experimental infants at their first exposure. Thus, the difference between the experimental and age-matched control groups is suggestive of physiologic based memory processes. Importantly, the changes in the cortisol response when infants of the experimental group were re-exposed after two weeks to maternal unresponsiveness were mediated by individual differences in physiological reactivity (i.e., *increasers vs. decreasers*). Thus, in the experimental condition the infants’ stress response in the second exposure appeared to have been triggered by recall of prior stress situation, albeit in different ways for the two reactivity groups.

Specifically, infants who showed a cortisol increase from pre-FFSF to post-FFSF in the first exposure, showed a cortisol decrease when re-exposed two weeks later to the maternal still-face. Conversely, infants who showed a cortisol decrease in the first exposure, showed a cortisol increase in the second exposure to maternal unresponsiveness. One possible explanation is that for *increasers* the decline in cortisol reactivity between first and second exposure reflects a familiarity effect recorded at the physiological level, whereby cortisol responses habituate to reiterated stressful event. Gunner and colleagues [44] demonstrated that newborn infants showed diminished cortisol reactivity to a physical examination when repeated 24 hours after a previous examination. For *decreasers* the rise in cortisol levels at the second exposure might indicate infants’ anticipatory response characterized by greater stress based on expectations about how their mothers will treat them in a specific context [13]. Taken together, these results support the interpretation that individual differences in cortisol response during a social stress affect infant memory in particular ways. Currently, the sources of these effects in the cortisol response associated with stress are not fully understood [35] and several factors such as infants’ experience of maternal sensitivity [29], [45], infant’s temperament style [46], genetic and epigenetic processes [47], [48] might be involved [36], [49].

Additionally, the brain regions involved in infant memory have been a topic of considerable recent research [3], [9], [50]. Adult and animal research have been demonstrated that emotional-episodic memory is mediated via hippocampus-dependent neural circuits, including amygdala and prefrontal cortex [51], [52] and that cortisol secretion modulates neural activity in these regions [53]. While we recognize that the present work offers only indirect evidence regarding the role of these brain regions in infant emotional memories, our findings lead us to the hypothesis that even in the early infancy, memory for social and emotional stressors might be associated to the development of a complex amygdala-hippocampus network. It is well established that stress effects on memory in humans mainly involve the hippocampus [18]. In addition, although evidence suggests in the first months of life in animal and humans hippocampus is not yet fully mature [54], research on rat pups has documented that specific early experience (i.e., handling *vs.* isolation) appears to have effects on hippocampal develop [55]. On the other hand, evidence from human adults and animal studies indicates that, given the connectivity between the amygdala and the hippocampus the amygdala modulates the consolidation of long-term explicit memories of emotionally arousing experiences by influencing the encoding and the storage of hippocampal-dependent memories [52], [53], [56]. While to our knowledge, there are no morphometric studies on human infants in the first months of life, developmental neuroimaging investigations on nonhuman primates shows that early in infancy the amygdala appears to develop more rapidly than the hippocampal formation [57]. Thus, neurodevelopment and functional data seem to support the hypothesis that even at early age the amygdala and hippocampus might be involved in emotional memory for social stress. Of course, further research is needed to evaluate this hypothesis.

Although infant negative engagement increased over the episodes of the FFSF at each exposure, contrary our expectation, there were no significant behavioral differences between *increasers* and *decreasers* over episodes or exposures. Furthermore, no correlations were found between behavioral response and salivary post-stress cortisol reactivity. This lack of linkage raises the issue of the connectedness between physiological and behavioral responses. Previous studies highlight only a limited association between behavioral and cortisol response [33], [58]. In a study using a learning and frustration procedure no significant correlations between cortisol levels and negative emotional expression emerged [59]. Moreover, other researchers have reported a lack of high correspondence among behavioral and physiologic reactivity measures during FFSF [29]. A possible explanation of this dissociation is that there is a substantial lag between the onset of the stressor (i.e., maternal still-face) and the peak cortisol response and the time resolution of behavioral response is faster than that of HPA-axis reactivity [60]. This could contribute to explain the limited covariation between negative emotionality and cortisol response. Globally, this suggests that behavioral and HPA-axis reactivity may have separate functional patterns [30], [41], [61], but how these patterns work together remains unclear.

It is necessary to note some limitations of the study. First, in the control condition we evaluated infants’ reactivity to and memory for the interaction only one time at 4 months and 15 days. A more complete experimental design also would have been to assess infants at 4 months of age in the same context but without the still-face manipulation (i.e., in a continuous face-to-face procedure). This approach would have insured that control infants experienced the same situational factors (e.g., coming at lab, seeing the room and experimenters two times, in a way similar to the experimental group). Nevertheless, it is noteworthy that the control group at their first and only exposure exhibited the same behavioral and cortisol reactivity as did the experimental group at T1. This evidence suggests the control group is useful in terms of providing an internal replication of the findings. Second, findings are based only on four month-old infants, but memory is likely to be moderated by the infant age. For example, older infants with more mature self- and other-directed regulatory behaviors might better modulate their levels of stress [62], so that they might not show the memory for the stressful social event after two weeks. A third limitation is that we examined only one recall interval. Thus, we cannot exclude that the infant memory for the stressful event could be also moderated by length of recall interval. Accordingly, one could be expect to find smaller effect sizes when infants experience a longer recall interval. Future work could more carefully address these effects varying both the infant age and length of recall interval.

Although further research is needed, the present study suggests that infants remember a social stress at a surprisingly early age showing a long-term memory persisting for at least 15 days. Indeed, the changes in the post-stressor cortisol response observed in the second exposure to FFSF seem associated with previous experience of social stress. Interestingly, infants’ memory observed in this study is apparent for an acute, clearly episodic social emotional event (i.e., maternal still-face) with only one distinctive cue (the yellow smock) rather than for a reiterated event where even stronger memory effects would be expected. Moreover, the findings suggest that the relationship between social stress and memory is related to individual differences in infant physiological reactivity. Although one would expect that chronic reiterated exposures to stressful events, such as neglect, parental mood disorders or traumatic events would have strong lasting memory effects, it will be important to take into consideration that the memorial effects of these experiences may be mediated by infants’ characteristic response to the stress. Indeed, individual differences in reactivity may aid in our understanding of the variation in effects of children exposed to chronic stressors. In sum, the current study expands our understanding of infants’ memory for a stress experienced during a “live” social interaction, adding knowledge to our comprehension of the interplay of physiological reactivity and long-term memory for real-life events in the first months of life.
